# Effects of Rapid Weight Loss on Judo Athletes: A Systematic Review

**DOI:** 10.3390/nu12051220

**Published:** 2020-04-26

**Authors:** Nemanja Lakicevic, Roberto Roklicer, Antonino Bianco, Diba Mani, Antonio Paoli, Tatjana Trivic, Sergej M. Ostojic, Aleksandra Milovancev, Nebojsa Maksimovic, Patrik Drid

**Affiliations:** 1 PhD Program in Health Promotion and Cognitive Sciences, University of Palermo, 90133 Palermo, Italy; lakinem89@gmail.com; 2 Faculty of Sport and Physical Education, University of Novi Sad, 21000 Novi Sad, Serbia; roklicer.r@gmail.com (R.R.); ttrivic@yahoo.com (T.T.); sergej.ostojic@chess.edu.rs (S.M.O.); nebojsam@uns.ac.rs (N.M.); 3 Sport and Exercise Sciences Research Unit, University of Palermo, 90133 Palermo, Italy; antonino.bianco@unipa.it; 4 Department of Applied Physiology and Kinesiology, University of Florida, Gainesville, FL 32611, USA; dmani@ufl.edu; 5 Department of Biomedical Sciences, University of Padova, 35122 Padova, Italy; antonio.paoli@unipd.it; 6 Faculty of Medicine, University of Novi Sad, 21000 Novi Sad, Serbia; lesandra_5@yahoo.com

**Keywords:** combat sports, martial arts, weight cutting, performance, judo athletes, health, psychological well-being

## Abstract

Rapid weight loss (RWL) is commonly practiced among judo athletes. Although it helps them to gain the advantage over their lighter opponents, previous studies have shown that RWL can have a negative impact on the athlete’s performance and overall well-being. This systematic review aimed to synthesize the evidence that examines the influence of rapid weight loss on physiological parameters, biomarkers, and psychological well-being in judo athletes. We followed the preferred reporting items for systematic reviews and meta-analyses guidelines. We searched for studies on Web of Science and PubMed that elaborate on the influence of ≥5% RWL achieved over ≤7-day period in judokas. Out of 52 studies initially found, 14 studies met our eligibility criteria and were included in the review. In total, we examined data from 1103 judo athletes. Retrieved studies showed conflicting data concerning physiological parameters and biomarkers, while psychological well-being parameters were more consistent than physiological and biomarkers. The feeling of tension, anger, and fatigue significantly increased while a decrease in vigor was demonstrated among athletes who lost weight rapidly. The evidence on the impact of RWL on performance remains ambiguous. More studies under standardized conditions are needed in order to provide firm evidence. Considering the harmful effects of RWL outlined in the existing literature, it is important to determine and monitor athlete’s minimal competitive weight to prioritize the health and safety of the athlete, emphasize fairness, and ultimately benefit the sport.

## 1. Introduction

Like other combat sports, judo is a weight categorized sport. Before each tournament, athletes undergo weight measurement to identify their competitive weight category. The weight control procedure was established in the past in order to give everyone a fair chance to compete with other individuals with similar characteristics [1]. In theory, athletes of the same anthropometric characteristics have comparable physical abilities and, as such, are suitable to compete in their given weight category. In the interest of gaining the edge over their opponents, many judokas (judo athletes) use well-known tactic of rapid weight loss (RWL) just before the competition. This behavioral pattern appears to be prevalent amongst judokas [2]. Some studies have shown that athletes who lose more weight during RWL have a great chance of success during combat [2,3,4,5,6]. Participants choose to be in the upper spectrum of their specific weight class for the sake of obtaining physical advantage over lighter opponents [7,8]. Thus, weight loss strategies have become a standard practice for those who engage in judo [5,9,10,11].

The literature shows that prevalence of RWL in martial arts ranging from 53% to 100% [9,12]. In a study completed by Artioli et al. [5], it has been shown that nearly 90% of both male and female judokas evaluated (*n* = 822) were using RWL techniques before official weigh-in sessions. The same study concluded that the most prevalent degree of weight loss was 5% of body weight, which was carried out over five days ahead of the competition [5]. Since the majority of judo athletes are usually unable to maintain their body weight within this weight class limit, they usually regain weight promptly after weigh-in [13], meaning it is needed to decrease it again for upcoming competitions [2]. Consequently, due to this pattern of weight loss and regain, combat athletes are considered “weight cyclers” [2]. On a broader scale, combat sport athletes usually eliminate approximately 2% to 10% of their body weight prior to every competition, mainly in the 2–3 days before the weigh-in session [2].

To achieve the desired weight, judo athletes use a wide range of methods that help them lose excessive weight quickly. Widespread weight loss techniques such as skipping meals and increasing exercise, restricting fluid intake, training with rubber suits, and sauna use are some of those methods practiced to reduce body mass prior to competition [7]. Beyond allowed RWL methods, pharmaceutical drugs are often used, although often labeled as illegal by competition committees [13]. Given the relevance of RWL strategies in judo athletes, this work aimed to systematically review the existing literature to examine which methods are most frequently used when inducing RWL in judokas and how RWL affects their physiological parameters, biomarkers and psychological well-being.

## 2. Materials and Methods

### 2.1. Literature Search Strategy

To ensure transparent and complete report, the preferred reporting items for systematic reviews and meta-analysis (PRISMA) guidelines were followed for conducting a systematic review [14].

EndNote software (Clarivate Analytics, Jersey, UK, ver. 8.1.) was used for the organization of content acquired by the article searches. Two electronic databases explored for article collection were Web of Science and PubMed. The following string was applied: “rapid weight loss” AND “judo”; “rapid weight loss” AND “combat sports”; “rapid weight loss” AND “martial arts”; “weight reduction AND judo”; “weight reduction AND combat sports”; “weight reduction AND martial arts”. 

**Figure 1:**
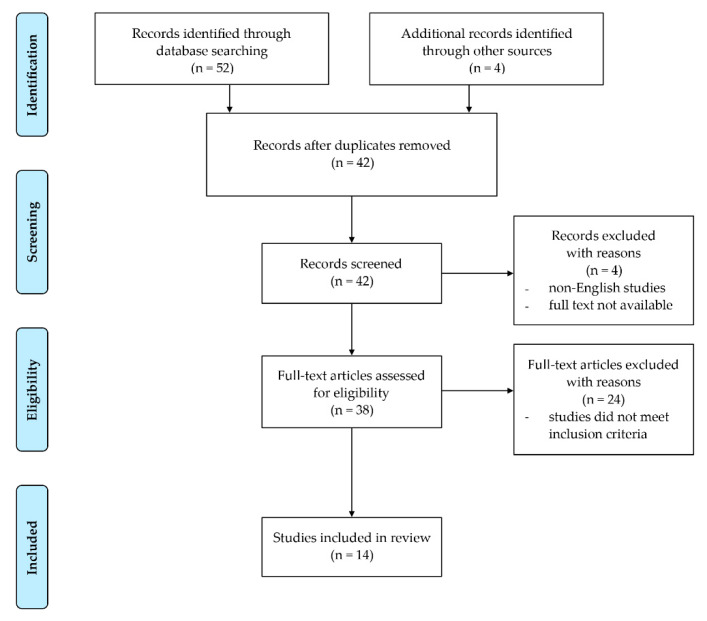
PRISMA flow diagram [14].

The article screening was carried out in a three-step procedure: title reading, abstract reading, and then full-text reading. If any disputes were shown between the two investigators, a third one considered the current process independently and discussed the decision with the other investigators. Notably, investigators were not blinded to the manuscripts, study title, authors, or associated institutions during the selection process. The screening processes have been summarized via the PRISMA flow diagram shown in Figure 1.

### 2.2. Inclusion and Exclusion Criteria

Only original articles written in English and published in peer-reviewed journals were considered for inclusion within this review. The date limit for publication period was set from the year 1996 until December 2019. Various formats of publications such as reviews, meta-analyses, abstracts, citations, scientific conference abstracts, opinion pieces, books, book reviews, statements, letters, editorials, nonpeer reviewed journal articles and commentaries have been excluded. There was no age limit for the participants and both genders were eligible to be included in the review. Eligible articles had to be conducted in judo athletes and had to include RWL within it. The extent of RWL had to be approximately 5% of their body weight regardless of the gender of participants. In addition, RWL had to be achieved over a period of up to seven days before weigh-in session. If a particular study involved illegal substances to elevate the magnitude of RWL, it was still included in the study for further analysis. Both qualitative and quantitative articles were taken into consideration.

### 2.3. Data Extraction

Critical information about included studies was delineated through tables (Microsoft Word 2007, Microsoft, Washington, USA) while a narrative description was performed to analyze the included literature on the topic. Certain specifics about a particular study that expand beyond tabular explanation have been described thoroughly in a narrative manner in the results section. Retrieved data acquired from included articles dealt with the influence of RWL on physiological parameters, biomarkers, and psychological well-being of judokas.

### 2.4. Risk of Bias Assessment

**Figure 2:**
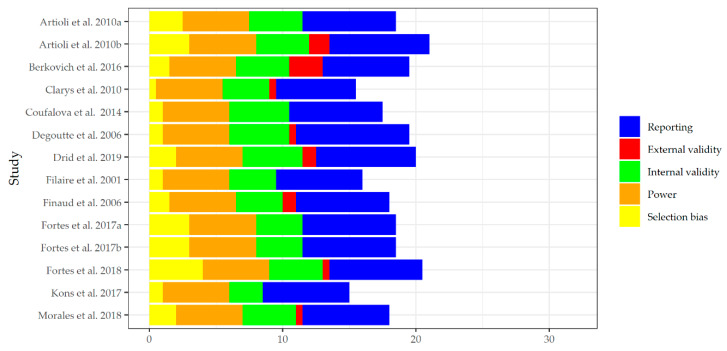
Risk of bias assessment [15].

The risk of bias assessment was carried out by two independent researchers through the Downs and Black checklist [15]. As suggested by Tremblay et al. [16], after assessment, studies were then separated into groups and labeled as ‘high quality’ (score 23–32), ‘moderate quality’ (score 19–22), ‘lower quality’ (score 16–18), or ‘poor quality’ (<14). Moreover, an average of all scores was calculated to determine the overall quality of the included studies as shown in Figure 2. The interclass correlation statistical method (SPSS, IBM, New York, USA, v.20) was used to determine inter-rater reliability between two researchers who completed the checklist. Quality of evidence was determined by the study design and the Downs and Black score.

## 3. Results

The preliminary title and abstract search revealed 52 articles through both PubMed and Web of Science. After the application of eligibility criteria on each article’s title and abstract, 28 articles were considered to be eligible for further examination. Additionally, four articles that were not found through our initial search but were thought to be relevant were added for full-text analysis. These four articles were suggested by the third researcher, who found them in the relevant bibliography. Duplicates were removed, leaving 26 articles to be reviewed in depth. Furthermore, two articles were removed due to lack of full-text availability (abstract only) and two other articles were removed due to non-English language use and no translation. Of the 22 articles analyzed, eight were excluded from the review due to lack of relevance or unavailability of relevant data. Therefore, a total number of 14 studies were included in this review [5,7,17,18,19,20,21,22,23,24,25,26,27,28], which were assessed for the methodological quality via the Downs and Black risk of bias checklist [15]. The average score for the included studies was 18.5 as shown in Figure 2. In accordance with the quality classification suggested by Tremblay et al. [16], the average score of included studies was labeled as moderate quality. Scores ranged from 13 to 22, with higher scores indicating better quality. Interclass correlation between two researchers assessing the risk of bias was 0.5, which can be considered to be moderate.

### 3.1. Sample Characteristics

In total, we examined data acquired from 1103 judo athletes (avg. age: 20.48 ± 3.22 yrs.; 5% of body weight loss; avg. RWL period: 6.5 days) within 14 studies that met our inclusion criteria as shown in Table 1. 

The most commonly practiced methods to induce RWL were found to be fluid intake reduction [5,7,18,19,20,22,23,26,27,28], caloric restriction [7,17,22,27], plastic suit training [5,7,20,23], increased physical activity [7,22,26], heated room training [7,20], sauna use [7,23], gradual dieting [7], spitting [5], and using laxatives [23]. In order to evaluate the effects of RWL on judokas’ well-being, the results have been summarized into three categories: (1) physiological parameters, (2) biomarkers, and (3) psychological well-being. All of the psychological measures were assessed via a profile of mood state questionnaire (POMS) [29] in all three studies examining the impact of RWL on psychological well-being.

### 3.2. Effects of Rapid Weight Loss on Physiological Parameters in Judokas

Five studies assessed the effects of RWL on strength as shown in Table 2 [17,18,21,22,28]. Degoutte et al. [18] examined a maximal isometric strength via hand-grip in the left hand, only. The subjects’ dominant hand was not reported. Two groups were tested on three different occasions: baseline, on the morning of the simulated competition, and ten minutes after a simulated competition. The experimental group showed a significant decrease in left hand-grip measurement prior to simulated competition, whereas the control group showed no difference within this period. After the competition, hand-grip values decreased in weight loss group, while the non-weight loss group showed a significant decrease compared to baseline and second measurement. Filaire et al. [17] also used hand-grip to determine isometric strength, but examined both hands. A significant decrease in the left hand-grip has been noted after RWL compared to the baseline measurements, while no differences were detected in the right hand-grip. In a study done by Coufalova et al. [22], isometric strength of arm, leg, trunk flexion and extension were measured as the peak force produced by maximal voluntary contraction at predefined positions. In addition, hand-grip was used to determine the isometric strength of hands. There were no significant differences in isometric strength except trunk flexion.

Clarys at et al. [21] observed the effects of RWL on strength in a high weight reduction group (≥3%) and low weight reduction group (≤3%). Isometric strength was not affected in the low weight reduction group, while in the high weight reduction group, the total isometric strength and isometric strength as measured during the second maximal effort of the 40-s method, decreased significantly. Morales et al. [28] assessed the isometric strength of hands through hand-grip and isometric trunk contraction for 3 s in a rapid (>3%) and progressive weight loss group (<3%). The results showed no significant interaction or main effect for either hand-grip or isometric trunk contraction in both groups.

Four studies assessed the effect of RWL on power [17,18,20,21]. Artioli et al. [20] examined the effect of RWL on upper-limb Wingate performance in the RWL group and a control group. Minimal improvement in performance has been observed after intervention in both groups compared to the baseline measurements. There was a significant main effect of time for relative and absolute mean power, peak power, and total work [20]. Moreover, no main group or interaction effects were found, implying that RWL did not affect performance. Clarys et al. [21] also measured anaerobic power in the high weight loss group (≥3%) and low weight loss group (≤3%) with a test comprising of five series of 20 maximal jump squats interspersed with 1-min rest periods. Average jump height was not affected by RWL as detected in both groups. Equally, no differences were found for ground contact times.

Filaire et al. [17] assessed vertical jumping height in two different modalities: without a preliminary countermovement jump and with a preliminary countermovement jump. The total flight time and the number of jumps performed were essential for the calculation of mechanical power. RWL did not influence simple jump and countermovement jumps. Furthermore, RWL did not impact total work output during the seven second test but induced a decrease during the 30 s test. Degoutte et al. [18] assessed anaerobic power of the upper limbs through the 30 s of horizontal isometric rowing in the weight loss and control group. The weight loss group showed a significant decrease in isometric strength compared to presimulated competition measurements, whereas the non-weight loss group showed significant decreased compared to baseline and morning of the simulated competition. 

Two studies assessed the influence of RWL on reaction time [21,28]. Clarys et al. [21] measured reaction time using an optical measurement system and software. Three repetitions were conducted with rest intervals of at least 15 s. Reaction time was not affected in the reaction tests for the low weight reduction group; a significantly shorter reaction time instead was found for the third attempt of the high weight reduction group when compared to baseline.

Morales et al. [28] measured reaction time with a contact platform connected to a microcontroller and a computer to observe acquired results. The experiment was conducted in the RWL group (>3%), progressive weight loss group (<3%), and control group. Reaction time was significantly higher post-test than pretest in the RWL group; no differences were found in the progressive weight loss and control groups.

One study assessed impact of RWL on balance. Morales et al. [28] evaluated balance from the center of pressure data that were collected using a balance board. The RWL group showed significant decreases in balance performance while judo athletes in the progressive weight loss and control group maintained balance performance. 

One study [23] assessed impact of RWL on decision making performance using the game performance assessment instrument (GPAI) [30]. The results showed that judokas who adopted RWL methods had no changes in the GPAI, while the control group showed a significant boost in performance. 

Another study also specifically assessed judo related performance in the RWL and control groups [30]. There was no group or time main effects or interaction effects for time structure patterns in the combats. Moreover, no significant differences between the groups in the number of attacks during judo combats were observed.

One study assessed the impact of RWL on the special judo fitness test (SJFT) in the RWL and control groups [24]. A heart rate monitor was used to register participants’ heart rate immediately following and 1-min after the test. Based on the number of throws performed and the heart rate values, a score was calculated. A significant group and time interaction effect was observed for the SJFT index, with a small improvement only for the control group. There was also a group and time interaction for total number of throws in the test, with a small increase in the control group and a small decrease in the weight loss group. There were no group, time or group, and time interaction effects for heart rate immediately after the test. Conversely, one minute after the test, there was a group and time interaction for heart rate, revealing a small increase for the weight loss group and a small decrease for the control group.

### 3.3. Effects of Rapid Weight Loss on Biomarkers

Four studies assessed the alterations in biomarkers induced by RWL, as shown in Table 3 [17,18,19,20]. Filaire et al. [17] examined the effect of RWL on serum total cholesterol (TC), triglycerides (TG), phospholipids (PHL), free-fatty acid (FFA), glycerol, low-density lipoprotein cholesterol (LDL-C), high-density lipoprotein cholesterol (HDL-C), alipoprotein (Apo), and alipoprotein A1 (Apo-A1). Notably, TG and FFA values significantly increased at the follow-up measurements, while the rest of the examined parameters remained unaffected.

Finaud et al. [19] investigated an RWL group and a control group, assessing biochemical parameters during three different time points: the weight maintenance period, seven days after the weight loss, the morning of a simulated competition, and ten minutes after the end of the competition. The assessed parameters included TG, FFA, glycerol, conjugated dienes, maximal rate of oxidation, lag phase and uric acid, where only conjugated dienes values remained unaffected at each time point measured. In the weight loss group, TG levels significantly decreased at the second time point compared to the baseline, and also 10 min after the end of the competition compared to second measurement. The control group presented decrements in TG levels only at the third measurement compared to the second. Free fatty acids values significantly increased at the second measurement compared to the weight maintenance period, whereas at the third measurement, the values decreased compared to the second. Changes through time points for FFA levels of the control group were the same as for TG. Glycerol values for the weight loss group increased only at the second measurement and returned to the stable range ten minutes after the competition. The control group demonstrated a significant increase in glycerol levels only at the third measurement, and those values were significantly higher compared to the second. The evolution of the rate of oxidation values decreased in the same manner for both groups. In fact, ten minutes after the competition, the assessed values significantly decreased compared to precompetition levels. The lag phase in the weight loss group significantly increased at the second measurement compared to the first and also increased at the third measurement compared to the second, with even greater significance. The control group demonstrated a significant increase in this parameter only ten minutes after the competition when compared to the second measurement. Uric acid concentration values changed in parallel to the lag phase values in both examined groups.

Degoutte et al. [18] adopted the same experimental design as Finaud et al. [19]. The assessed biomarkers were TG, FFA, glycerol, ammonia, uric acid, urea, glucose, alkali reserve, adrenocorticotropic hormone (ACTH), cortisol, testosterone, testosterone/cortisol ratio, dehydroepiandrosterone sulfate (DHEA-S), DHEA-S/C, insulin, and thyroid hormones ratio. Plasma levels of TG, FFA, glycerol, and uric acid changed over time in the same fashion as described by Finaud et al. [19]. Ammonia levels significantly increased at the third measurement in the weight loss and control groups compared to the second measurement. Urea concentration in the weight loss group increased at the second measurement compared to the baseline values and was significantly raised at the third measurement when compared with the second. The control group showed an increase in urea levels only at the third time point compared to the second.

Interestingly, glucose values significantly increased only for the weight loss group at the third measurement compared to the second. Within the control group, the levels of glucose remained stable over time. Alkali reserve values presented a significant decrease ten minutes after the competition in both the weight loss group and control group, whereas data for the baseline values were not reported in this study.

Increases in ACTH levels were demonstrated only by the weight loss group in the morning before the competition in comparison to the baseline values, while the control group showed no changes over time. Cortisol and ACTH values altered in a same fashion for the weight loss group and remained stable for the control group across timepoints. The weight loss group demonstrated testosterone level decreases at the second measurement compared to the baseline, as well as at the third measurement compared to the second. The values of testosterone in the control group significantly dropped only ten minutes after the competition in comparison to precompetition values. The testosterone/cortisol ratio changed in a same fashion as testosterone, with a higher level of significance at the second measurement for the weight loss group and at the third measurement for the control group. Dehydroepiandrosterone sulfate values significantly increased at the second measurement for the weight loss group, while no significant changes were observed at the third measurement. The levels of this hormone in the control group remained unchanged throughout the study. The dehydroepiandrosterone sulfate/S ratio significantly decreased at the second measurement in the weight loss group, while the changes in the control group were not observed throughout timepoints.

The weight loss group presented a significant decrease of insulin after the weight-loss period, while the level of this hormone increased significantly ten minutes after the competition compared to the second measurement. In the control group, values of insulin were significantly raised only at third compared to second measurement. The testosterone/cortisol ratio dropped significantly at the second measurement; nevertheless, it increased at the third measurement with the same level of significance in the weight loss group. For the control group, these values remained unchanged throughout the protocol. 

Artioli et al. [20] followed plasma lactate and plasma glucose levels in the RWL and control groups at two different time points set five days apart. Plasma lactate did not differ between the groups and the values remained stable throughout the study. 

In a study conducted by Drid et al. [27], serum creatinine levels in male judokas significantly increased after seven days of RWL procedure, while guanidinoacetic acid and creatine values remained unaffected.

### 3.4. Effects of Rapid Weight Loss on the Psychological Well-Being in Judokas

Psychological well-being of judokas has been assessed in three studies as shown in Table 4 [17,18,25]. In each investigation, the POMS questionnaire was used. The authors presented the impact of RWL on six different mood states: tension, depression, anger, vigor, fatigue, and confusion. Filaire et al. [17] tested eleven athletes who used RWL methods to achieve a target weight within one week. Participants had to complete the questionnaire before and after the RWL period. According to the Filaire et al. [17], a significant increase in fatigue and anger was found at the follow up assessment. In addition, tension and confusion also increased with even higher levels of significance compared to the baseline values. A decrease was noticed in vigor, while depression remained unchanged among judokas. Degoutte et al. [18] assessed the psychological well-being of two groups at three different time points, as described earlier.

A tension increase was noticed at the second measurement for weight loss group, as well as at the third measurement compared to second. The control group demonstrated increased tension only at the third measurement when compared to second. Feelings of anger were significantly higher in the morning of the simulated competition compared to the baseline for the weight loss group. However, values returned to be insignificant ten minutes post competition. The control group demonstrated no significant alterations in assessed timepoints. Vigor among the weight loss group decreased significantly when measured after the RWL period. Values of vigor were still decreased at the third measurement compared to the second. In the control group, vigor decreased only at the third measurement compared with the second. The impact of seven-day RWL on fatigue was noticed at the second measurement in the weight loss group, where the values were significantly lower than at first measurement. Ten minutes after the end of the competition, fatigue was still elevated compared to the second measurement among this group. A significant decrease in fatigue was present only at the third measurement compared to the second when the control group was evaluated. In this study, no alterations were observed in depression and confusion in either of the groups at each timepoint.

Fortes et al. [25] assessed how the POMS were affected by a two-week RWL (10% weight loss) and control group. The POMS questionnaire had to be completed by both groups at the beginning and after the protocol. Both groups demonstrated an increased feeling of tension at post measurements. The control group showed no significant difference in terms of depression at either time points, while judokas in the weight loss group reported greater levels of depression during the follow-up assessment. In terms of vigor, an increase in the control group at post measurement was observed, although the weight loss group demonstrated a significant decrease after the weight reduction assessment. A state of confusion remained unaffected in both groups.

## 4. Discussion

### 4.1. Overall Summary

Studies addressing the effects of RWL on physiological parameters have produced conflicting data, with negative [17,21,22,25,28], positive [20,28], and negligible [17,20,21,22,28] effects being demonstrated. The majority of studies that measured hand-grip strength reported similar outcomes [17,22,28]. Generally, hand-grip strength after RWL was not impaired but neither improved. Two studies that measured reaction time reported different results [21,28]. However, Clarys et al. [21] reported a decrease in reaction time during the third measurement in a high weight reduction group while a low weight reduction group revealed unchanged values in all three measurements.

On the other hand, Morales et al. [28] revealed improved reaction time in the group that lost weight rapidly. Jumping abilities among judokas appeared to be unaffected by RWL [17,21]. Sport-specific performance tests seemed not to be affected by RWL procedures, which have not had a significant impact on sport-specific performance [23,24]. 

In terms of biomarkers alterations, no distinctive trend could be observed. However, in studies where two different groups were included, distinctive changes could be noticed in follow-up measurements of the experimental group [18,19,20]. Furthermore, two authors reported an increase in FFA levels at follow-up measurements [17,19]. Except for the mentioned studies, no distinctive trend has been reported. 

Since all authors used the POMS scale for psychological well-being assessment, it can be noticed that RWL similarly affected judokas’ psychological state in all studies that assessed psychological well-being [17,18,25]. The feeling of tension, anger, and fatigue increased significantly, while a decrease in vigor was demonstrated among athletes who lost weight rapidly.

### 4.2. Strengths and Limitations

Our study is not without limitations. Although we have thoroughly searched the existing literature on RWL and its impact on physiological parameters, biomarkers and psychological well-being, we found relatively few studies. Furthermore, we found a high methodological heterogeneity amongst included studies, and this might be one of the major reasons for controversial results, which precludes our ability to draw firm conclusions. Some of the study designs did not include a control group and thus limited authors from examining crucial variables concurrently. However, in the scarcity of available studies, we still included these studies given the inclusion of important parameters that were in the experimental groups. Included studies also varied vastly in sample size, variables measured, and age of the participants. 

Strengths of this review embedded a comprehensive search strategy, stringent predetermined inclusion and exclusion criteria and thorough analyses of each article included. Due to narrow scope of this review coupled with strict inclusion–exclusion criteria, it was expected that we would not find many studies that meet our criteria. As a result, we selected only 14 articles which were included in the review with a sum of 1103 participants. At the beginning of the review planning, we wanted to conduct a more detailed meta-analysis that would have allowed us to estimate the overall effect sizes for the desired outcome. However, a meta-analysis was precluded due to the high heterogeneity of the data. Therefore, key findings were reported narratively. Based on the data acquired through our systematic literature search, we were unable to conclusively outline clear cut evidence, except for the psychological well-being parameters which all drawn from the studies that used the same test battery. 

### 4.3. Mechanisms Affecting Performance During Rapid Weight Loss

Considering the prevalence of RWL in judo, it seems reasonable to address the negative aspects associated with RWL primarily in terms of performance and health. According to American College of Sports Medicine, there is a general consensus in the literature that RWL has a negative impact on physiological and health-related parameters [31]. Moreover, negative psychological consequences induced by RWL have been reported in multiple studies [17,18,32,33], which might affect athletic performance. 

Despite heterogenic evidence investigating the effect of RWL on performance, most studies indicate that RWL impairs both aerobic and anaerobic performance [13]. While aerobic performance detriments have been assigned to dehydration, lower plasma volume, elevated heart rate, hydroelectrolytic fluctuations, defective thermoregulation and muscle glycogen depletion [34], decline in anaerobic performance is primarily related to reduced buffering capacity, glycogen depletion, and hydroelectrolytic alterations [34,35]. In addition, RWL can cause perturbations in normal homeostatic functions of the endocrine system [36].

Although there is scarce evidence investigating the specific dietary approaches athletes implement to induce RWL [37], commonly seen acute caloric restriction is likely to influence performance through depleted glycogen depots [38]. Reduced levels of muscle glycogen have been shown to cause muscle fatigue by impairing the excitation–contraction link in the muscle tissue [39]. This could negatively impact enzymes related to anaerobic glycolysis [40,41], which could undermine high-intensity exercise performance [37,42], as seen in judo [43]. The exercise intensity during the judo matches to about 92% of maximal oxygen consumption [44]. The intermittent nature of judo, with multiple matches within 24 h, requires an optimization of muscle glycogen storage, which is compromised during periods of low carbohydrate intake, dehydration, and intense training [33,45]. According to Greenhaff et al. [46], even with sufficient food intake, a low carbohydrate diet may impair the buffering capacity of the blood. The coupled effects of RWL and a low carbohydrate intake would down-regulate the muscle hydrogen ion efflux, which can accelerate fatigue during intense muscular contractions [17].

Dehydration prompted through excessive sweat leads to a reduction in the blood plasma and therefore total blood volume is also reduced, hampering cardiovascular function, muscle blood flow, and thermoregulatory ability [47,48]. Plasma volume changes might also combine with the detected decline in hemoglobin mass following RWL to impair aerobic capacity [8,49]. Moreover, reduced plasma volume results in reduced cardiac output and increased blood viscosity [2], which impedes normal blood flow to the working muscles. Isacco et al. [50] recorded the heart rate of male judokas during fights after a RWL period and: the average was ~174 bpm and, with maximal ~187 bpm, which represents 92% and 99% of their theoretical maximal heart rate, respectively. Thus, it can be concluded that a drastically elevated heart rate is a compensation for a reduced stroke volume caused by reduced blood volume. 

Short-term hypohydration initiated by RWL may considerably shift electrolyte concentration, which can influence the cellular fluid homeostatic control, its metabolic processes, and as a result, damage neuromuscular efficiency [37,51,52]. Some studies further indicate that dehydration impacts mental fatigue by increasing the rate of perceived of exertion during exercise and negatively influencing mood-state [37,53,54,55].

Regarding the impact of RWL on the endocrine system, a positive correlation has been found between the dehydration level and cortisol concentration, whereas a negative correlation was observed between dehydration and total testosterone [56]. However, an acute increase in the cortisol level can be considered as a normal occurrence during intense exercise [57,58]. Other potential risks associated with RWL include endocrine disturbances, insulin sensitivity alterations, bone degradation, and impaired immune function [37,41,57,59]. In particular, hormonal disturbances induced by repetitive RWL have been shown to negatively affect normal growth and development in adolescence [13,60]. Importantly, there is now substantial data suggesting that athletes who engage in RWL at early ages are at higher risk of weight loss-related problems long-term [2,13,61].

Finally, some studies have related RWL with increased risk for injuries [62]. In a study by Oopik et al. [63], it was found that the 5% reduction in body mass affected metabolism and muscle contraction patterns, thereby elevating the risk of injury. Green et al. [64] also demonstrated that judokas who practiced RWL that eliminated more than 5% were more prone to injury during competition in comparison to judokas who lost less than 5% of their body weight.

### 4.4. Final Remarks

Based on the hazardous effects associated with RWL, in 2016, Artioli et al. [2] suggested banning RWL in combat sports. Prohibiting RWL procedures would prioritize the health and safety of the athlete, emphasize fairness, and ultimately benefit the sport [2]. We strongly agree with claims posed by Artioli et al. [2] since, by now, there is enough data to suggest that RWL can affect safety and athletes’ health and well-being both acutely and chronically. The problem of RWL came to the light when it was discovered that a 5-year old boy was engaged in RWL [65] and when three National Collegiate Athletic Association (NCAA) wrestlers died of hyperthermia when trying to lose 15% of their body weight before the competition [66]. Franchini et al. [13] suggested for combat athletes to rather engage in gradual weight loss at the rate of about one kilogram per week, while implementing regular resistance exercise to preserve muscle mass. Franchini et al. [13] also added that carbohydrate intake should be emphasized and that most of the weight loss should be coming from fat substrates. Emerging evidence has shown that intermittent fasting can be a way to promote healthy weight loss while maintaining muscle mass [67]. In terms of regulations, Franchini et al. [13] suggested that matches should start in an hour or less after weigh-ins and that athletes should be required to pass a hydration status test when weighing in. In addition, we propose regular weight screening (i.e., on a quarterly basis) for all combat sport athletes where, consequently, their minimal competitive weight can be determined in the beginning of the season. Since RWL is commonly practiced in other combat sports, a systematic review of the literature regarding impact of RWL in boxing, wrestling, taekwondo, etc. can be carried out to determine similarities and differences between its impact on judo and other combat sports.

## 5. Conclusions

The impact of RWL on strength and power remains equivocal, while no distinctive trend besides increased free fatty acid mobilization has been observed within the investigated biomarkers. This may indicate increased level of lipolysis due to glycogen depletion. With respect to the impact of RWL on psychological well-being parameters, the feelings of tension, anger, and fatigue significantly increased, while a decrease in vigor was demonstrated among athletes who lost weight rapidly. More research is needed to access the overall influence of RWL on judo athletes’ performance and well-being. However, considering the harmful effects of RWL outlined in the existing literature, it is important to determine and monitor athlete’s minimal competitive weight to prioritize the health and safety of the athlete, emphasize fairness, and ultimately benefit the sport.

## Figures and Tables

**Table 1:** Study design and participants’ characteristics.

Author	N		Age (years)	RWL period (weeks)	Baseline (kg)		Follow-up (kg)	Weight lost (%)			RWL methods
	EG	CG									
Filaire et al. (2001) [17]	11		NA	7 days	75.1 ± 2.6		71.5 ± 1.3	~5 (4.8)			-Caloric restriction
Degoutte et al. (2006) [18]	10	10	NA	7 days	75.9 ± 3.1		72.1 ± 1.4; 74.5 ± 3.4*	~5			-Caloric restriction; fluid intake reduction
Finaud et al. (2006) [19]	10	10	NA	7 days	75.9 ± 3.1		72.1±1.4; 74.5 ± 3.4*	~5			-Caloric restriction; fluid intake reduction
Artioli et al. (2010) [20]	7	7	21 ± 4	5 days	77.9 ± 12.2		74.1 ± 11.4	5			-Fluid intake reduction; plastic suit training; heated room training
Artioli et al. (2010) [5]	822		19.3 ± 5.3	7 ± 7 days	70 ± 7.5		NA	2–5			-Fluid intake reduction; plastic suit training; spitting
Clarys et al. (2010) [21]	22		21.8 ± 3.8	5 ± 3	72.0 ± 12.3		69.0 ± 11.7	≥3			NA
Coufalova et al. (2014) [22]	9		22.3 ± 2.44	5 day	79.14 ± 8.97		75.74 ± 9.51	4.6			-Caloric restriction; increased physical activity
Berkovich et al. (2016) [7]	108		14.6 ± 1.6	8 ± 5.4 days	58.0 ± 12.1		55.3 ± 10.6	~5			-Fluid intake reduction; caloric restriction; increased physical activity; plastic suit training; heated room training; sauna; gradual dieting
Kons et al. (2017) [26]	12		23.3 ± 5.5	7 days	3 months	1 week	82.92 ± 21.73	≤5			-Fluid intake reduction; increased physical activity
					87.21 ± 21.84	85.49 ± 22.3					
Fortes et al. (2017) [23]	20	19	22.2 ± 1.8	14 days	72.5 ± 3.6		64.8 ± 4.0	10			-Fluid intake reduction; plastic suit training; sauna; laxatives
Fortes et al. (2017) [24]	20	19	22.2 ± 1.8	14 days	72.5 ± 3.6		64.8 ± 4.0	10			-Fluid intake reduction; plastic suit training; sauna; laxatives
Fortes et al. (2018) [25]	20	19	22.2 ± 1.8	14 days	72.5 ± 3.6		64.8 ± 4.0	10			-Fluid intake reduction; plastic suit training; sauna; laxatives
Morales et al. (2018) [28]	38		20.6 ± 2.6	7 days	M & F (CG, PWL, RWL)		M & F (CG, PWL, RWL)	CG	PWL	RWL	-Fluid intake reduction
					66.5 ± 13.1		NA	NA	<3	>3	
Drid et al. (2019) [27]	8		19.3 ± 2.0	7 days	81.7 ± 10.7		76.8 ± 10.3	6			- Fluid intake reduction; caloric restriction

Abbreviations: M—male; F—female; CG—control group; PWL—progressive weight loss; RWL—rapid weight loss; WS—weight stable group; WL—weight loss group; NA—not available; *—regained weight.

**Table 2:** Effects of rapid weight loss on physiological parameters in judokas.

Study	Test	Results											
Filaire et al. (2001) [17]	Performance tests	T1								T2			
	RHG	↔								↔			
	LHG	↔								↓			
	SJ	↔								↔			
	CMJ	↔								↔			
	7s jumping	↔								↔			
	30s jumping	↔								↓			
Degoutte et al. (2006) [18]		T1			T2					T3			
		WL	CG		WL		CG			WL		CG	
	Grip strength	↔	↔		↔		↔			↔		↓(vsT1&vsT2) (*p* < 0.01)	
	30s isometric horizontal rowing	↔	↔		↔		↔			↔		↓(vsT1&vsT2) (*p* < 0.01)	
Clarys et al. (2010) [21]		HWRG								LWRG			
	Reaction time (ms)	Rt1 ↔ Rt2 ↔ Rt3 ↓								Rt1↔ Rt2↔ Rt3↔			
	Max. Isometric contraction (N)	Isomax0 ↓ Isomax1 ↓ Isomax2 ↓ Isomax3 ↓ IsomaxT↓								Isomax0 ↔ Isomax1 ↔ Isomax2 ↔ Isomax3 ↔ IsomaxT ↔			
	Mean jump height (cm)	Mjh1↔ Mjh2↔ Mjh3↔ Mjh4↔ Mjh5↔								Mjh1↔ Mjh2↔ Mjh3↔ Mjh4↔ Mjh5↔			
Artioli et al. (2010) [20]	Wingate performance	EG↑								CG↑			
	Judo combat (number of attacks)	EG↔								CG↔			
Coufalova et al. (2014) [22]	Changes in maximal isometric strength	Hand grip(R&L)								↔			
		Arm flexion (R&L)								↔			
		Arm extension (R&L)								↔			
		Trunk flexion								↓			
		Trunk extension								↔			
		Knee flexion (R&L)								↔			
		Knee extension (R&L)								↔			
Fortes et al. (2017) [23]	GPAI (game performance assessment instrument)	EG						CG					
		pre		post				pre			post		
		↔		↔				↔			↑		
Fortes et al. (2017) [24]		EG						CG					
		pre		post				pre			post		
	SJFT Number of throws (n)	↔		↓				↔			↑		
	Heart rate (bpm)	↔		↑				↔			↓		
Morales et al. (2018) [28]		Balance			Reaction time					Iso. strength			
		CG	PWL		RWL	CG	PWL		RWL	CG		PWL	RWL
	Performance tests	↔	↔		↓	↔	↔		↑	↔		↔	↔

Abbreviations: RHG—right hand grip strength; LHG—left hand grip strength; SJ—squat (simple) jump; CMJ—counter movement jump; 7s jumping—7 s jumping test; 30s jumping—30 s jumping test; T1—baseline; T2—follow up; Rt—reaction time; Isomax—maximal isometric strength; Mjh—mean jump height; HWRG—high weight reduction group; LWRG—low weight reduction group; EG—experimental group; CG—control group; PWL—progressive weight loss group; RWL—rapid weight loss group; Iso.strength—isometric strength; SJFT—Special Judo Fitness Test; pre—baseline measurement; post—follow-up measurement; WL—weight loss group; CG—control group; T1—baseline period; T2—morning of a simulated competition; T3—10 min after the end of the competition [18]; ↔—no significant change; ↑—significant increase; ↓—significant decrease.

**Table 3:** Effects of rapid weight loss on biomarkers.

Author	Tests	Results								
Filaire et al. (2001) [17]	Biomarkers	T1					T2			
	TC (mmol·L^−1^)	↔					↔			
	TG (mmol·L^−1^)	↔					↑^*^			
	Ph - lipid(mmol·L^−1^)	↔					↔			
	FFA (mmol·L^−1^)	↔					↑^*^			
	Glycerol (mmol·L^−1^)	↔					↔			
	LDL –C (mmol·L^−1^)	↔					↔			
	HDL –C (mmol·L^−1^)	↔					↔			
	Apo (g·L^−1^)	↔					↔			
	Apo- A1 (g·L^−1^)	↔					↔			
	B/A1	↔					↔			
Finaud et al. (2006) [19]		T1			T2			T3		
		WL	CG		WL	CG		WL		CG
	TG (mmol·L^−1^)	↔	↔		↓^*^	↔		↓^#^		↓^#^
	FFA (mmol·L^−1^)	↔	↔		↑^*^	↔		↓^#^		↓^#^
	Glycerol (μmol·L^−1^)	↔	↔		↑^*^	↔		↔		↑^##^
	CDmax (UA)	↔	↔		↔	↔		↔		↔
	Rmax (UA)	↔	↔		↔	↔		↓^##^		↓^##^
	Lp (min)	↔	↔		↑^*^	↔		↑^##^		↑^#^
	Uric acid (μmol·L^−1^)	↔	↔		↑^*^	↔		↑^#^		↑^##^
Degoutte et al. (2006) [18]		T1			T2			T3		
		WL	CG		WL	CG		WL		CG
	TG (mmol·L^−1^)	↔	↔		↓^*^	↔		↓^#^		↓^#^
	FFA (mmol·L^−1^)	↔	↔		↑^*^	↔		↓^#^		↓^#^
	Glycerol (μmol·L^−1^)	↔	↔		↑^*^	↔		↔		↑^##^
	Ammonia (μmol·L^−1^)	↔	↔		↔	↔		↑^#^		↑^##^
	Uric acid (μmol·L^−1^)	↔	↔		↑^*^	↔		↑^#^		↑^##^
	Urea (mmol·L^−1^)	↔	↔		↑^*^	↔		↑^#^		↑^##^
	Glucose (mmol·L^−1^)	↔	↔		↔	↔		↑^#^		↔
	Alkali reserve (mmol·L^−1^)	NA	NA		↔	↔		↓^#^		↓^##^
	ACTH (pg·mL^−1^)	↔	↔		↑^*^	↔		↔		↔
	Cortisol (mmol·L^−1^)	↔	↔		↑^*^	↔		↔		↔
	Testosterone (nmol·L^−1^)	↔	↔		↓^*^	↔		↓^#^		↓^#^
	Testosterone/Cortisol	↔	↔		↓^**^	↔		↓^#^		↓^##^
	DHEA-S (μmol·L^−1^*)*	↔	↔		↑^*^	↔		↔		↔
	DHEA-S/C	↔	↔		↓^**^	↔		↔		↔
	Insulin (mUI·L^−1^)	↔	↔		↓^*^	↔		↑^##^		↑^##^
	T3/T4	↔	↔		↓^*^	↔		↑^#^		↔
Artioli et al. (2010) [20]		Baseline				Follow up				
		WL		CG		WL			CG	
	Plasma glucose (mg·dL^−1^)	↔		↔		↓			↔	
	Plasma lactate (mM)	↔		↔		↔			↔	
Drid et al. (2019) [27]	Serum:	Baseline				Follow up				
	Creatine (μmol/L)	↔				↔				
	Creatinine (μmol/L)	↔				↑^*^				
	Guanidinoacetic acid (μmol/L)	↔				↔				

Abbreviations: *—*p* < 0.05; **—*p* < 0.01 (T2 vs. T1); TC—serum total cholesterol; TG—triglycerides; Ph-lipids—phospholipids; FFA—free fatty acids; LDL-C—low-density lipoprotein cholesterol; HDL-C—high-density lipoprotein cholesterol; Apo(A1/B)—apolipoprotein (A1/B); T1—baseline; T2—follow-up [17]; *—T_2_ vs. T_1_ (*p* < 0.05),** —(*p* < 0.01); #—T_3_ vs. T_2_ (*p* < 0.05); ##—T_3_ vs. T_2_ (*p* < 0.01); WL—weight loss group; CG—control group; TG—triglycerides; FFA—free fatty acid; CDmax—maximum amount of conjugated dienes; Rmax—evolution of maximal rate of oxidation; Lp—length of lag phase; ACTH—Adrenocorticotropic hormone; DHEA-S—Dehydroepiandrosterone sulfate; DHEA-S/C(ratio); T3/T4—thyroid hormones; T1—baseline period; T2—morning of a simulated competition; T3—10min after the end of the competition; NA—not available; [18,19]; *—*p* < 0.01 [27]; ↔—no significant change; ↑—significant increase; ↓—significant decrease.

**Table 4:** Effects of rapid weight loss on the psychological well-being in judokas.

Author	POMS Questionnaire	Results							
Filaire et al. (2001) [17]		T1				T2			
	Tension	↔				↑^**^			
	Depression	↔				↔			
	Anger	↔				↑^*^			
	Vigor	↔				↓^*^			
	Fatigue	↔				↑^*^			
	Confusion	↔				↑^**^			
Degoutte et al. (2006) [18]		T1			T2		T3		
		WL	CG		WL	CG	WL		CG
	Tension	↔	↔		↑^*^	↔	↑^#^		↑^#^
	Depression	↔	↔		↔	↔	↔		↔
	Anger	↔	↔		↑^**^	↔	↔		↔
	Vigor	↔	↔		↓^**^	↔	↓^#^		↓^#^
	Fatigue	↔	↔		↑^**^	↔	↑^#^		↑^#^
	Confusion	↔	↔		↔	↔	↔		↔
Fortes et al. (2018) [25]		CG				WL			
		pre		post		pre		post	
	Tension	↓^*^		↔		↓^*^		↑^#^	
	Depression	↔		↔		↓^*^		↑^#^	
	Anger	↔		↔		↓^*^		↑^#^	
	Vigor	↓^*^		↔		↑^*^		↓^#^	
	Fatigue	↔		↔		↓^*^		↑^#^	
	Confusion	↔		↔		↔		↔	

Abbreviations: *—*p* < 0.05; **—*p* < 0.01 T2 vs. T1 [17]; *—T_2_ vs. T_1_ (*p* < 0.05), **—(*p* < 0.01); #—T_3_ vs. T_2_ (*p* < 0.05); ##—T_3_ vs. T_2_ (*p* < 0.01); WL—weight loss group; CG—control group; T1— baseline period; T2—morning of a simulated competition; T3—10min after the end of the competition [18]; pre—pretest measurements; post—post-test measurements; *—*p* < 0.05 in relation to the post-test; #—*p* < 0.05 in relation to the CG in the post-test [25]; ↔—no significant change; ↑—significant increase; ↓—significant decrease
